# Microbial Primer: Bacterial DNA supercoiling

**DOI:** 10.1099/mic.0.001667

**Published:** 2026-02-13

**Authors:** Charles J. Dorman

**Affiliations:** 1 Department of Microbiology, Moyne Institute of Preventive Medicine, Trinity College Dublin, Dublin D02 PN40, Ireland

**Keywords:** DNA supercoiling, DNA topoisomerases, transcription, twin supercoiling domain model

## Abstract

DNA in most bacterial cells is maintained in an underwound state. The DNA double helix responds to underwinding by adopting a minimum energy conformation through the supercoiling of the duplex, the formation of local single-stranded bubbles or a combination of both. This Microbiology Primer summarizes the key topological features of DNA and describes the topoisomerase enzymes that manage bacterial DNA topology. The influences of variable DNA topology on transcription and of transcription (and DNA replication) on DNA topology are also discussed. Finally, the article considers the impact of changes in bacterial metabolism and physiology on DNA topology and their implications for bacterial pathogenesis.

## Introduction

**Fig. 1.**
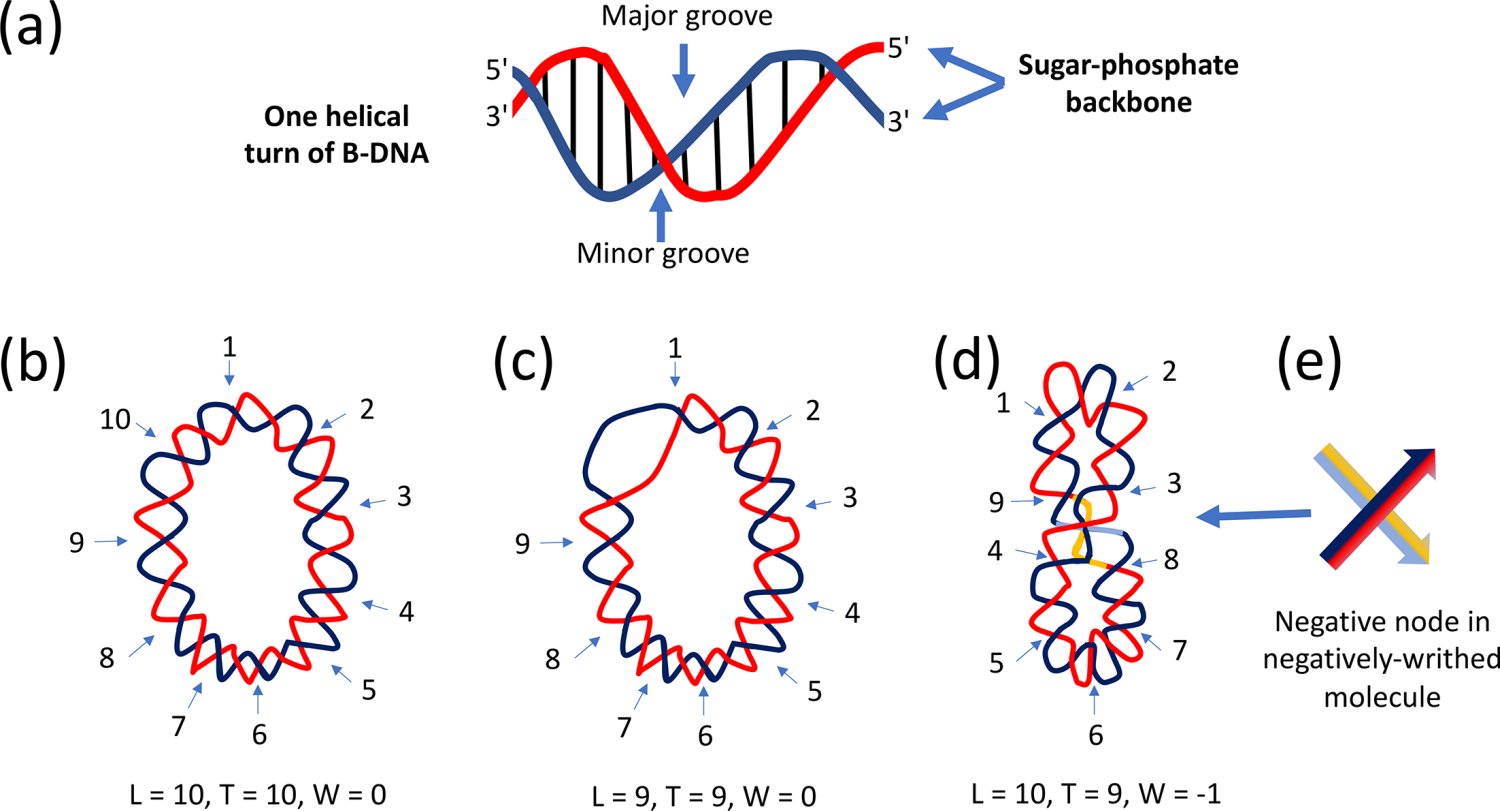
Fundamental elements of DNA structure and topology. (**a**) One helical turn of B-DNA is illustrated. The sugar-phosphate backbones of the DNA duplex are represented by the red and the blue curves; the vertical lines are the base pairs that connect them. The major and minor grooves are indicated by vertical arrows. (**b**) A relaxed, covalently closed, circular DNA molecule is shown. It contains 105 bp and 10 helical turns. The linking number (L) of the molecule is 10, its twist number, T, is also 10 and it contains no writhing turns (W=0). Because the circle is relaxed (or free from torsional stress), it lies in a flat plane. (**c**) This circular DNA molecule is a topological isomer of the molecules shown in panels (**b**) and (**d**). Its linking number has been reduced by 1 (ΔL=−1; L=9). The molecule experiences torsional stress due to the linking number reduction, and this is resolved by the formation of a bubble of ssDNA, where one helical turn is removed (ΔT=−1; T=9). This minimum energy conformation allows the circle to lie in a flat plane without the adoption of a writhed topology (W=0). (**d**) Here, the underwound circle has responded to the linking number deficit (ΔL=−1) while maintaining full base-pairing (T=10). Torsional stress has been resolved by the adoption of a writhed structure (W=−1), and the molecule no longer lies in a flat plane. This writhing turn of the duplex axis is equivalent to one negative supercoil. (**e**) The crossed arrows illustrate the pathways of the dsDNA negative node shown in panel (**d**) with the dark blue and red strands passing over the pale blue and yellow ones.

The discovery that DNA has an antiparallel, double-stranded helical structure [1] was a foundational event in modern genetics and molecular biology. An analysis of the DNA duplex reveals not only how it stores genetic information in the sequence of its purine and pyrimidine bases but also how that information can be copied with high fidelity during DNA replication. The antiparallel arrangement of the DNA strands arises because one strand runs in the 5′-to-3′ direction and the other in the 3′-to-5′ direction (Fig. 1a). The sugar-phosphate backbones of the DNA strands are on the outside of the double helix, imparting to it the appearance of smooth structural uniformity. Structural diversity lies within the duplex where the chemically distinct A, C, G and T bases are located. Hydrogen bonds between the bases hold the two strands together: A-T (or T-A) pairs have two hydrogen bonds, while C-G (or G-C) pairs have three. The bonds are broken when the interwound strands of the duplex undergo transient, localized separation during gene expression or DNA replication to give the transcription or replication machineries access to the base sequence. This strand separation has consequences for the higher-order structure of the DNA. Proteins involved in transcription regulation and/or the imposition of structural adjustments to the DNA can gain access to the bases through the grooves in the duplex surface (Fig. 1a).

## DNA topology

This article will focus on circular B-DNA, the form that is most commonly found in bacterial cells. Each strand in B-DNA executes one complete turn around the other every 10.5 bp (Fig. 1a). The two strands may be regarded as coiling around an invisible central axis whose path describes the trajectory of the DNA duplex through space. The topology of the duplex is described using three parameters, L, T and W (in some publications, these parameters are written, respectively, as L_k_, T_w_ and W_r_) (Fig. 1b–e). The linking number, L, is an integer that describes the number of times one DNA strand crosses over the path of the other. The twist number, T, is the number of complete rotations, or turns, of the strands around the duplex axis. The writhe number, W, is the number of times the central axis turns around itself. Axial writhing (Fig. 1d) approximates to one’s intuitive understanding of 'supercoiling' – it involves the coiling of a duplex molecule whose constituent strands are already coiled around one another. Writhing can occur in a negative sense (as seen in underwound DNA) or in a positive sense when DNA is overwound. It is these isomeric forms of DNA writhing that give rise to negative DNA supercoiling and positive DNA supercoiling, respectively [2,3].

The inter-relationships of the three parameters are as follows:

L=T+W   (1)

A change to one parameter in equation (1) is usually distributed between the other two:

ΔL = ΔT + ΔW   (2)

For example, equation (2) predicts that if the number of times one DNA strand coils around the other is increased (over-twisting) or decreased (under-twisting), this will be reflected in a change to the linking number, a change to the writhe of the duplex or a change to both (Fig. 1c, d). L_0_ is the linking number of a fully relaxed DNA circle. If this DNA molecule becomes underwound, it acquires a new L value that is less than L_0_ (L < L_0_). Overwinding the DNA causes L to exceed L_0_ (L > L_0_). Circular dsDNA molecules with identical base sequences but different topologies are called topoisomers. Changes to L require the breakage and reunion of one or both strands of the DNA, and the enzymes that perform these reactions are called topoisomerases.

## Topoisomerases

**Fig. 2.**
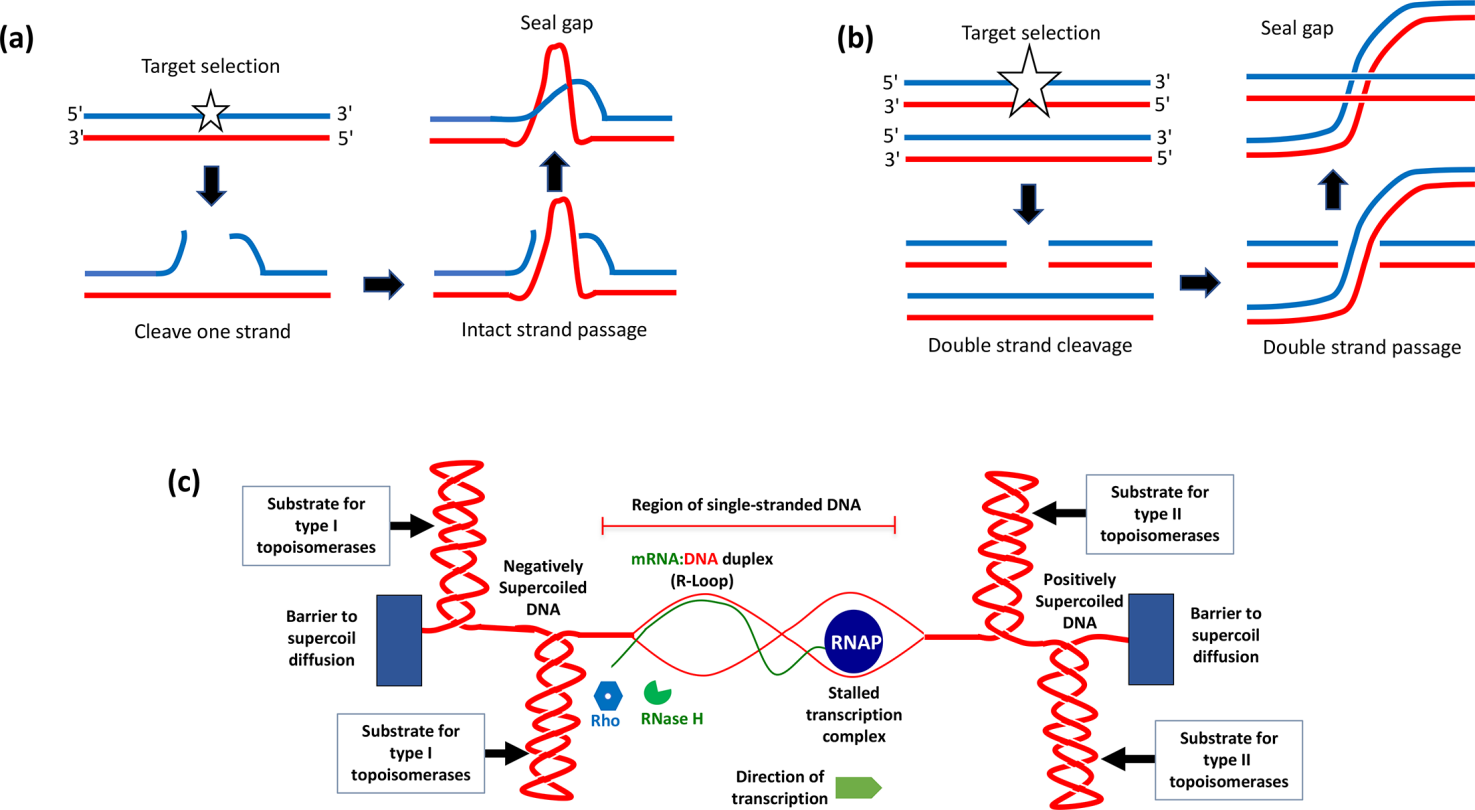
Changes to DNA topology arising from topoisomerase activity and local distortions associated with transcription. (**a**) Topo I cleaves the top strand (blue) of the DNA duplex and passes the intact (red) strand through the resulting gap before resealing it. This changes the linking number of the DNA by +1. (**b**) DNA gyrase makes a double-stranded break in DNA and passes part of the intact duplex through the gap before resealing it. The linking number of the DNA is changed by −2. (**c**) A stalled transcription complex is shown with a positively supercoiled DNA domain ahead and a negatively supercoiled domain behind. Barriers prevent domain dissipation, so topoisomerases must relax the supercoils. Transcript base-pairing with its DNA template creates R-loops if the RNA is not first degraded by RNase H. Ribosome-free RNA in the stalled elongation complex may allow the Rho factor to terminate transcription prematurely.

A comprehensive review of topoisomerase diversity in bacteria and higher organisms is beyond the scope of this article, but an excellent introduction to the topic may be found in McKie *et al*. [4]. We will use *Escherichia coli* K-12 as our model bacterium, which has four topoisomerases. These are DNA topoisomerase I (topo I), DNA gyrase, DNA topoisomerase III (topo III) and DNA topoisomerase IV (topo IV) (Table 1). Topos I and III are type I enzymes that change L in steps of one; gyrase and topo IV are type II enzymes that change L in steps of two. Topo I in *E. coli* is a type IA enzyme that relaxes negatively supercoiled DNA by a single-strand passage mechanism (Fig. 2a). The enzyme binds to DNA and makes a 5′-ssDNA nick, forming a covalent bond to one side of the resulting gap (Fig. 2b). It then passes the intact DNA strand through the opening and re-seals the gap, changing the linking number of the DNA by +1. Gyrase is a type IIA enzyme that introduces negative supercoils into DNA via a reaction in which both strands of the DNA are broken, an intact portion of the duplex DNA is passed through the opening and the gap is resealed (Fig. 2b). ATP hydrolysis is required for the completion of the reaction, and the DNA’s linking number change is −2. Gyrase uses the same ATP-dependent reaction to remove positive supercoils from DNA. In addition, it can relax negatively supercoiled DNA without ATP hydrolysis. Topo III is a decatenase and is concerned with the smooth operation of chromosome segregation at cell division; topo IV also plays a crucial role in daughter chromosome segregation (Table 1).

**Table 1.** The topoisomerases of *E. coli*

Topoisomerase	Type	Gene(s)	Subunit structure	Activities
DNA gyrase	IIA	*gyrA gyrB*	Tetrameric: A_2_B_2_	Double-strand passage through DNA gap. ATP-dependent introduction of negative supercoils/removal of positive supercoils. ATP-independent DNA relaxation. ATP-dependent catenase/decatenase.
Topo I	IA	*topA*	Monomeric	Single-strand passage through 5′-ssDNA gap. ATP-independent relaxation of negatively supercoiled DNA. ATP-independent catenase/decatenase.
Topo III	IA	*topB*	Monomeric	Single-strand passage through 5′-ssDNA gap. ATP-independent relaxation of negatively supercoiled DNA. ATP-independent catenase/decatenase.
Topo IV	IIA	*parC parE*	Tetrameric: A_2_B_2_	Double-strand passage through DNA gap. ATP-dependent relaxation of negatively or positively supercoiled DNA. ATP-dependent catenase/decatenase.

## Topoisomerases, transcription and DNA replication

The movements of transcription complexes and DNA replication forks each cause local topological distortion of the DNA template. In the case of DNA-dependent RNA polymerase, its translocation during transcription produces a build-up of over-wound (or positively supercoiled) DNA ahead and a local domain of under-wound (or negatively supercoiled) DNA behind (Fig. 2c). These events, originally predicted in an insightful theoretical paper [5], have since been confirmed experimentally in both prokaryotic and eukaryotic systems [6].

Rotation of the bulky transcription complex around the DNA does not occur with sufficient efficiency to resolve the topological impediment to further transcript elongation. This problem is exacerbated in the case of mRNA production due to the association of ribosomes with the transcript. If the downstream positive supercoils do not diffuse laterally along the DNA, due to a diffusion barrier such as a nucleoprotein complex or an oncoming transcription complex on the opposite DNA strand, transcription will stall (Fig. 2). A stalled transcription complex may facilitate the generation of an R-loop if the nascent RNA message base-pairs with its complementary DNA strand in the hyper-negatively supercoiled DNA that has formed in the wake of the transcribing RNA polymerase (Fig. 2c). R-loops can provoke hyper-recombination and hence DNA damage. Thus, they present a threat to the integrity of the genome and to the viability of the cell.

Topo I prevents R-loops from forming by eliminating the negative supercoils that are generated in the wake of transcription elongation complexes; should an R-loop arise, its RNA component is degraded by RNase H (Fig. 2c). DNA gyrase relaxes the positive supercoils that occur ahead of the elongation complex, allowing the complex to continue its forward movement, reducing the likelihood that R-loops will form upstream of the complex. The same principles apply in the case of a moving DNA replication fork: Topo I and gyrase intervene to maintain smooth traffic flow along the DNA template.

## DNA supercoiling and gene expression

A bubble of ssDNA is formed during the assembly of the transcription initiation complex at gene promoters. The torsional stress produced due to the underwound nature of negatively supercoiled DNA might be expected to assist bubble formation by facilitating the breakage of hydrogen bonds between bases. The degree of torsional stress required varies from promoter to promoter due to local issues of promoter structure. Local negative supercoiling of DNA at the promoter can be generated by DNA gyrase, by the divergent transcription of an upstream gene (with concomitant underwinding of the DNA behind RNA polymerase) or as a result of DNA underwinding in the wake of a passing DNA replication complex.

Sensitivity to variations in DNA topology is not restricted to the initiation step in transcription: elongation and termination are also sensitive. DNA topological influences on elongation have been described in the preceding section. If the topological barriers to free movement of RNA polymerase are not resolved, transcription will either stall (often accompanied by RNA polymerase back-tracking) or terminate prematurely. Termination is driven in G+C-rich DNA by the Rho termination factor. The hexameric Rho protein binds and translocates along ribosome-free RNA to make contact with RNA polymerase, displacing the transcript from the transcription complex (Fig. 2c).

## DNA topology and cellular energetics

The ATP-dependent type II topoisomerases of *E. coli* are sensitive to the ATP/ADP ratio in the cell, which reflects cellular metabolic status [7]. For this reason, the topological profile of the genome mirrors the stage that the bacterium has reached in its growth cycle. Not to be confused with the cell cycle, the growth cycle describes the journey of a bacterial batch culture from inoculation through the principal stages of growth: lag, log and stationary phase. (Each phase may be subdivided into early, middle and late, with stationary being followed by a death phase.) We will consider an aerated *E. coli* batch culture growing in a defined medium with glucose as a carbon source at 37 °C. The lag phase is a period of adjustment during which the inoculum (typically a small volume of a starter culture that has been grown overnight) moves from stationary phase to the onset of exponential growth. Each cell must assemble the machinery needed to exploit its new environment. The DNA in the starter culture reflects that of an energy-poor stationary-phase bacterium – it is topologically relaxed, a state that correlates with a low ATP/ADP ratio. The ratio increases as the pool of ATP in the cell expands as the bacterium imports glucose while respiring aerobically. Using reporter plasmids or other probes of DNA topology to monitor genomic topology, the DNA of the cell will be seen to become more supercoiled compared to the lag-phase culture. This mirrors shifts in the transcriptome and the modulation of type II topoisomerase activities, as well as structural adjustments to the genome imposed by architectural components such as nucleoid-associated proteins. In lay terminology, the economy of the bacterium is humming, the currency of exchange (ATP) is abundant and one of the indices of this strong growth is a shift in DNA topology from a relaxed state to one of negative supercoiling [6].

The culture grows exponentially as long as the carbon source, other essential nutrients and the oxygen supply are maintained. In batch culture, an exponential expansion of the culture is not sustainable indefinitely, and so bust eventually follows boom. The metabolic slump is reflected in a slowing of growth, a decline in the ATP/ADP ratio and a shift in DNA topology towards a relaxed state. This transition is accompanied by a transcriptomic transition as many exponential-phase genes become silent while others become transcriptionally active [7]. The latter group is made up of the stationary-phase genes, many of which are transcribed by a re-programmed RNA polymerase that contains the RpoS protein, a sigma factor that replaces the housekeeping RpoD sigma factor that predominated in exponential phase. The RpoS form of RNA polymerase is more tolerant of a relaxed DNA template than RpoD [8]. It is important to emphasize that the exponential-to-stationary growth-phase shift is complex and involves many more factors than just a DNA topological transition and the recruitment of an alternative sigma factor by RNA polymerase [9]. Growth cessation can be imposed at any time by the application of a physical or chemical shock. Experiments with bacteria carrying reporter plasmids have shown that growth-arresting stress is accompanied by changes to the plasmid topoisomer distributions. Because bacterial pathogens experience physical and chemical environmental stresses during the infection of their hosts, it is unsurprising that sensitivity to variable DNA supercoiling is a feature of many of the genes that pathogens rely on to initiate and to maintain an infection [10].

## Abbreviations

A: adenine
C: cytosine
G: guanine
L_0_: the linking number of a relaxed DNA circle
L/L_k_: linking number
T: thymine
topo I: topoisomerase I
topo III: topoisomerase III
topo IV: topoisomerase IV
T/T_w_: twist number
W/W_r_: writhe number
